# Growing old at home – A randomized controlled trial to investigate the effectiveness and cost-effectiveness of preventive home visits to reduce nursing home admissions: study protocol [NCT00644826]

**DOI:** 10.1186/1471-2458-8-185

**Published:** 2008-05-28

**Authors:** Steffen Fleischer, Gudrun Roling, Katrin Beutner, Stephanie Hanns, Johann Behrens, Tobias Luck, Bettina Kuske, Matthias C Angermeyer, Steffi G Riedel-Heller, Sven Heinrich, Hans-H König, Christine Lautenschläger

**Affiliations:** 1 Institute of Nursing and Health Science, Medical Faculty, Martin-Luther-University Halle-Wittenberg, Germany; 2 Public Mental Health Research Unit, Department of Psychiatry, University of Leipzig, Germany; 3 Center for Public Mental Health, Gösing a.W., Austria; 4 Health Economics Research Unit, Department of Psychiatry, University of Leipzig, Germany; 5 Institute for Epidemiology, Biostatistics and Medical Informatics, Medical Faculty, Martin-Luther-University Halle-Wittenberg, Germany

## Abstract

**Background:**

Regarding demographic changes in Germany it can be assumed that the number of elderly and the resulting need for long term care is increasing in the near future. It is not only an individual's interest but also of public concern to avoid a nursing home admission. Current evidence indicates that preventive home visits can be an effective way to reduce the admission rate in this way making it possible for elderly people to stay longer at home than without home visits. As the effectiveness and cost-effectiveness of preventive home visits strongly depends on existing services in the social and health system existing international results cannot be merely transferred to Germany. Therefore it is necessary to investigate the effectiveness and cost-effectiveness of such an intervention in Germany by a randomized controlled trial.

**Methods:**

The trial is designed as a prospective multi-center randomized controlled trial in the cities of Halle and Leipzig. The trial includes an intervention and a control group. The control group receives usual care. The intervention group receives three additional home visits by non-physician health professionals (1) geriatric assessment, (2) consultation, (3) booster session.

The nursing home admission rate after 18 months will be defined as the primary outcome. An absolute risk reduction from a 20% in the control-group to a 7% admission rate in the intervention group including an assumed drop out rate of 30% resulted in a required sample size of N = 320 (n = 160 vs. n = 160).

Parallel to the clinical outcome measurement the intervention will be evaluated economically. The economic evaluation will be performed from a society perspective.

**Discussion:**

To the authors' knowledge for the first time a trial will investigate the effectiveness and cost-effectiveness of preventive home visits for people aged 80 and over in Germany using the design of a randomized controlled trial. Thus, the trial will contribute to the existing evidence on preventive home visits especially in Germany.

## Background

It can be stated as an obvious fact that people prefer to age at home in a familiar environment then to move to a nursing institution. Considering the aim of an autonomous and independent life as a central aspect of successful living in the older population, measures to achieve this aim have to be taken.

In modern industrialized countries traditional forms of support for the elderly are often replaced by new formal and informal support systems. This shift happened as a consequence of demographic and social changes in the 19th and 20th century. Concerns about the well-being of the elderly population grew especially in the second half of the 20th century as problems arose to afford for the care of the growing number of elderly people. In addition to the mentioned structural changes a reduced commitment to care for elderly family members is forecasted in industrialized countries.

Data from the German Federal Statistical Office indicates a total prevalence of people aged 80 and over that are cared for in nursing homes of 13% in the relevant German population [1]. A survey in Leipzig showed that about 27.8% of the population in nursing homes is between 80 and 84 years and even 60.8% is 85 years and older [2]. Relevant reductions of self care skills were found in 61.8% of the people 80 years or older [2]. To give concise prognosis of necessary nursing care 3 main factors have to be taken into account:

(1) Demographic developments (declining familial potential for nursing),

(2) Structural changes in society (increased female employment; increasing number of single-person households) and

(3) Cultural changes (decreasing commitment to nurse relatives).

Some authors extrapolate a 60% increase in the prevalence of people in need of long term care assuming a moderate increase in average life expectancy within the next 30 years [3], and assuming a higher increase in average life expectancy the increase would be even higher [3,4]. Consequently the need for inpatient care will rise accordingly. Inpatient care like residential care homes or nursing homes causes higher social costs than ambulatory care. An absolute or relative increase in admission rates thus would put a higher strain on the long term care insurance system in Germany [5,6]. Efficient measures to reduce the prevalence of nursing home admissions by either avoiding or delaying admission have to be investigated.

Preliminary models of preventive home visit interventions were developed in Denmark and the United Kingdom [7]. More then twenty years after the first intervention programs the concept of preventive home visits has been developed to multidimensional assessments accompanied by multidimensional interventions. As care for the elderly includes several dimensions these dimensions are represented in contemporary home visit programs. They include the socioeconomic supportive dimension, the social integrative dimension and finally the health dimension. The individual assessment of these dimensions is meant to identify individual risk factors or a decline of functioning, leading to a need for individual intervention strategies. The concept of home visits has the advantage to assess functioning in an individual's relevant environment [8,9]. In the most recent meta-analysis [10] 26 trials investigating preventive home visits were identified. The included trials were conducted in 8 different countries but Germany was not included. Results regarding the effectiveness of preventive home visits are discussed very controversially within the reviews and a strong clinical heterogeneity can be stated across the existing publications [9,10]. As such complex interventions may be highly sensitive to the local health system the generalizability of international results to Germany is limited. For that reason it is important to develop and investigate a concept of preventive home visits in Germany that accommodates local structures.

The aim of our study therefore is to investigate the effects of preventive home visits in Germany in people aged 80 and older. Our hypothesis is: Preventive home visits will reduce the incidence of nursing home admissions within the investigated period of 18 months. Furthermore we expect the intervention to be cost-effective. The results can be used as a basis for recommendations on the funding and implementation of preventive home visits in Germany.

## Methods

This multi center trial is based on a non-blinded randomized controlled study design. We have 2 centers included in this trial, Halle (Saxony-Anhalt) and Leipzig (Saxony). The control group receives usual care besides the baseline and the final measurement after 18 months while the intervention group additionally receives a geriatric assessment, a consultation visit and a booster session.

### Inclusion and exclusion criteria

The study is conducted in cooperation with the university hospital Halle, the Diakoniekrankenhaus Halle, the registration office Halle and 3 GP practices in the city of Halle and 18 GP practices in the city of Leipzig. All people older than 80, fluent German speakers, residents of Leipzig or Halle, living at home or planned discharge to home (hospital patients) are eligible for this study. Additionally, participants have to be impaired in at least 3 activities of daily living. We exclude from trial participation people that are cognitive impaired, not able to give informed consent or have a care level higher than I (according to German long term care insurance).

### Recruitment of participants

It is a well documented problem to recruit participants in this age group especially when interventions that demand home visitation are involved [11]. We therefore will implement three strategies to maximize recruitment and reach the needed sample size. The primary recruitment strategy is via GP practices in the cities of Halle and Leipzig. As a second strategy the recruitment via liaison nurses will be implemented in hospitals in Halle to cope with the small number of participating GPs and to include a group of high risk participants. As a third recruitment strategy people aged 80 and over in Halle and Leipzig will be contacted by mail. Addresses will be retrieved by cooperating with the local registration offices. We will send explicit trial information, a consent form for participants and a self-addressed stamped envelope. After return of the consent form we will contact the responders by telephone, prescreen for inclusion and exclusion criteria and make an appointment for a home visit. This first home visit is different to the first home visits of the other recruitment methods so far that the screening is done within that visit in addition to the assessment. Prescreening and final screening is necessary as we expect a certain amount of inappropriate responders to our letter. We will set a time limit of 4 weeks after dispatch of the last letter till we close participant registration via mail. All received consent letters then will be put in random order. This will be the order we contact the persons until we got enough study participants in addition to general practitioners' practices and hospital recruitment. Exceeding persons will get a written cancellation due to numerical limitation for trial participants.

### Randomization

A balanced block-wise randomization, stratified by center, will be used. In Halle the sample additionally is stratified according to the recruiting hospital and practice. Concealment of allocation is assured by central randomization via consecutive randomization lists in the order of recruitment.

Participants recruited via the registration office will be randomized using sealed opaque envelopes immediately after completion of the baseline assessment.

### Blinding

Patients and field researchers cannot be blinded to group allocation. A blinded statistician not involved in trial conduct will do the final analysis.

### Study procedure

A liaison nurses screens and recruits the participants for eligibility in the 2 hospitals in Halle. Medical secretaries screen and recruit the participants in Leipzig and Halle for eligibility in the participating GPs' practices. Patients then are randomized by the local study center via consecutive randomization lists in this way assuring concealed allocation of participants. The participants are contacted by the centers' study personal via mail and phone. In the phone call an appointment for the first home visit is made with the patients. In Halle we additionally will contact the patients after discharge from hospital. All other study procedures are standardized across the study centers, in this way ensuring a homogenous conduct of the trial.

The intervention group will be assessed with a geriatric assessment and the baseline assessment. A team of nurses, psychologists, physiotherapists, dietitians and physicians will work out individualized recommendations in a case review one week after the geriatric assessment. The main aim is to identify self-care deficits and risks for self-care deficits in the socioeconomic supportive dimension, the social integrative dimension and finally the health dimension. Then appropriate recommendations will be given by the team. After the case conference the members of the intervention group are visited by the same staff member again. This staff member will conduct a consultation and instruction based on the results of the case conference. Four weeks after the consultation another visit is done by the staff member. This visit (booster session) is for evaluative purposes mainly: To what extent the study participant adheres to the recommendations and how convenient are the recommendations. The final measurement will be the follow-up assessment 18 months after baseline.

**Figure 1:**
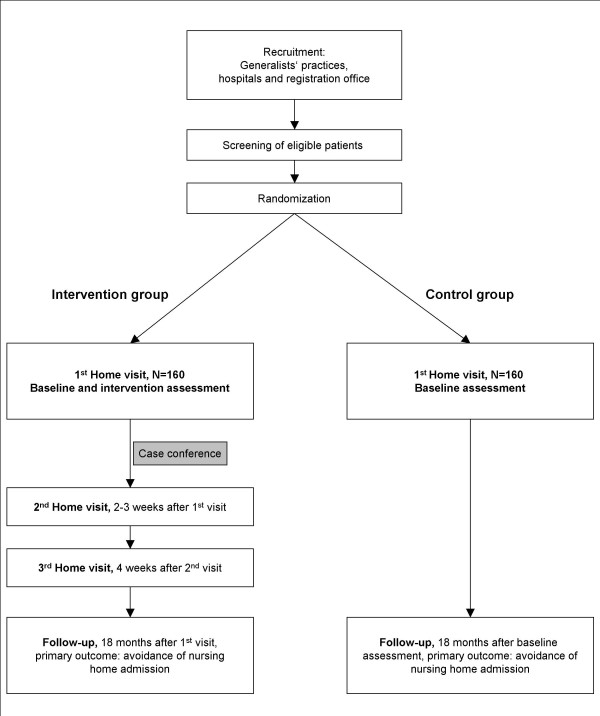
Study design.

The control group is only assessed with the baseline assessment and the follow-up assessment 18 months later. The control group will not receive any intervention in addition to standard care. The flow of the participants is shown in Figure 1.

### Outcomes

Primary outcome of our trial is the incidence of nursing home admission over the study period of 18 months. Nursing home admission in our trial is defined as the permanent admission into an inpatient nursing care facility according to the social security code of Germany. Short term care, assisted living, geriatric day care or respite care facilities are not included in this definition.

Data will be collected on follow-up assessment either by direct contact with the participants, their relatives or the resident registration office.

Secondary outcomes over the whole trial period of 18 months:

• Time to nursing home admission

• health care service utilization and costs

• incremental cost-effectiveness and cost-utility ratio

• health related functioning

• health related quality of life

• prevalence of falls

### Sample size calculation

Sample size calculation was based on an assumed absolute risk reduction from a 20% in the control group to a 7% in the home admission rate of the intervention group. Power calculation (α = 0.05; β = 0.20) including an estimated drop out rate of 30% resulted in a required sample size of N = 320 (n = 160 vs. n = 160) using a two-tailed Chi-square test. The sample size is equally divided among the two study centers Halle and Leipzig.

### Drop-outs

Drop-outs will be documented thoroughly and included in data analysis to the point of drop-out. Reasons for drop-out will be reported and analyzed.

### Data analysis

Clinical data analysis will be done by the institute for epidemiology, biostatistics and medical informatics of the medical faculty Martin-Luther-University Halle-Wittenberg.

In a first step adequate descriptive statistics will be used to compare the intervention group against control group after randomization. Then all outcomes will be tested in comparison of the intervention and control group with multifactorial regression models on an intention-to-treat basis. Level of significance is determined by 5%. Two-tailed tests for significance will be used for all statistical tests.

Precision of results will be specified. Confidence intervals (95%) will be computed for all primary and secondary outcomes.

Economic analysis will be conducted by the Health Economics Research Unit, University of Leipzig. This involves calculating costs as well as the cost-effectiveness ratio, i.e. the ratio of the difference in mean costs and the difference in mean effects between the intervention and control group. QALYs based on EQ-5D [12] will be used to measure health effects in cost-utility analysis. To assess the uncertainty of the results sensitivity analysis will be performed and cost-effectiveness acceptability curves will be computed.

### Quality assessment

The trial is part of the Nursing Research Network "Mitte-Süd". A report system is established within the network. Annual quality reports have to be prepared for the German Federal Ministry of Education and Research.

As the conduct of a multi center trial demands high standards of quality to warrant comparable conditions and results among the centers all procedures were developed and documented in joint commissions.

### Screening

Screening includes sociodemographic data (age, sex, marital status and housing conditions), information about long term care insurance, nursing allowance and degree of impairment to receive benefits from the long term care insurance. Furthermore, Activities of Daily Living (ADL) are checked [13].

### Baseline assessment

All participants (intervention and control group) receive baseline assessment after randomization and the same assessment at follow-up after 18 months. Cognitive function is assessed using the Mini Mental State Examination (MMSE) [14]. The health related quality of life is measured by the EuroQol-5D [12], including a visual analogue scale (VAS) to measure patients preferences. Psychosocial factors are assessed with the Social Situation by Nikolaus (SoS) [15]and the 5-item version of the Geriatric Depression Scale (GDS)[16]. This short form was chosen because it is as effective as the 15-item version of the GDS [17], which is widely used for depression screening in cognitively unimpaired elderly persons. Moreover, the 5-item GDS is more an economical version because of reduced administration time. Details about the functional status will be determined using the Barthel-Index [18] and the Instrumental Activities of Daily Living (IADL) [19]. Additionally, all participants are asked about their history of falls in the past 12 months. A Questionnaire of Service Utilization and Costs will be used based on cost diaries used in earlier studies [20-25].

This questionnaire also includes sociodemographic data (i.e. age, sex, marital and educational status, life conditions); information about health insurance, long term care insurance and the degree of impairment to receive benefits from the long term care insurance. Furthermore the Chronic Disease Score will be calculated [26].

### Intervention assessment

All subjects of the intervention group additionally receive intervention assessment immediately after baseline assessment. The current nutrition status is measured by the Mini Nutrition Assessment (MNA) [27]. Other health dimensions of elderly persons such as impaired sight or hearing, urinary or bowel incontinence and loss of functional muscle mass are assessed with the Geriatric Screening AGAST [28] and the Geriatric Screening by Lachs [29]. These geriatric screenings also determine social activities, housing conditions, economical conditions and polypharmacy. The Clock-Completion Test [30] will complement the MMSE for the baseline assessment of cognitive abilities.

### Case conferences

We will conduct interdisciplinary case conferences for all patients in the intervention group. We intend to collect several cases for a conference. We will conduct case conferences in the defined period of 3 weeks between first and second home visit in this way trying to standardize the procedures. Cases will be prepared and introduced to the conference group by the visiting investigator. The investigators will invite appropriate health professionals as the case requires. The professional spectrum of the conference expert advisory group hereby consists of nurses, a general practitioner, a nutritionist, a geronto psychiatrist, a physiotherapist, a psychologist and a social worker. Recommendations will be worked out for the single patient and we will document them for the next home visit. The duration of the preparation, present experts and duration of the conference will be documented for each patient individually.

### Home counseling intervention

The last part of the experimental intervention consists of a counseling intervention. We will address the identified problems and present the recommendations of our expert advisory group during this home visit. We estimate the duration of the second home visit including the counseling with 20–40 minutes.

### Booster session

Four weeks after the counseling home visit we will visit the patients in the intervention group for a third time. During this visit we want to evaluate which recommendations the patients already implemented and which not. In this way we can assess obstacles and facilitators to the recommendation adherence. Additionally, we have the opportunity to boost our recommendations from the second home visit.

### Follow-up

18 months after the first home visit we will assess all participants using the same measurement as for the baseline assessment. Additionally, details about current residence status, nursing home admission and date of nursing home admission are included. If a subject cannot be reached by usual means like telephone or mail, the named contact person or residents' registration office will be contacted.

All measurements are summarized in Table 1.

**Table 1:** Measurements

Points of measurement and outcome measures according to case report form	
**point of measurement**	**outcome measures**

Screening	▪ sociodemographic data
	▪ ADL – Activities of Daily Living [13]
	▪ document analysis

Baseline assessment	▪ EQ-5D [12]
	▪ Chronic Disease Score [26]
	▪ Number of falls during the last 12 months
	▪ Questionnaire of Service Utilization and Costs [20-25]
	▪ Barthel-Index [18]
	▪ MMSE – Mini Mental State Examination [14]
	▪ SoS – Social Situation [15]
	▪ 5-item version of the Geriatric Depression Scale [16]
	▪ IADL – Instrumental Activities of Daily Living [19]

Intervention assessment	▪ MNA – Mini Nutrition Assessment [27]
	▪ Clock-Completion Test [30]
	▪ Geriatric Screening AGAST [28]
	▪ Geriatric Screening by Lachs [29]

Follow-up	▪ same as Baseline

### Protection of data privacy

We will create a pseudonym for all trial participants to collect and analyze the trial data. Key lists will be stored separately from the trial data and erased after final data analysis. Data will be analyzed in a way that no conclusions can be drawn to individual participants. Trial data is stored in lockable cabinets in lockable rooms. All gained address data of persons not included in the trial will be erased after exclusion (prescreening or screening) or non-inclusion (non-responders of the registration offices' samples).

### Ethical considerations

The study protocol is approved by the ethics committees of the universities in Halle and Leipzig. If changes to the study procedures are necessary they will be proposed to the local ethics committees as amendments. All changes will be described and discussed in the publication of the trial's results.

### Publication policy

We plan to publish the trial results in a peer reviewed international Medline-listed journal whether the effectiveness was shown or not. This mainly serves the purpose to avoid publication bias. Additionally, we are obliged by the Federal Ministry of Education and Research to report our results within 6 months after study termination. All trial results will be reported within context to this study protocol.

## Discussion

A significant reduction in nursing home admission rates over the trial period is expected as a primary outcome. The study design of a randomized controlled trial is appropriate for the underlying research question. We deem the two recruitment approaches hospital and general practitioner's practice to be of practical relevance for the German health system. Assuming positive results general practitioners, hospitals or home care agencies seem a promising way to implement such an intervention in the German health system.

## Competing interests

The authors declare that they have no competing interests.

## Authors' contributions

SGR–H and JB are responsible project coordinators in the two participating study centers and the main investigators. SGR–H, JB, MCA, StH, SH, H–HK and BK were responsible for general study design. CL planned the statistical analysis, conducted the sample size calculation and was responsible as biometric counselor. H–HK and SH are responsible for economic evaluation. GR, KB, TL and SF are responsible for the conduct of the trial, amendments to the first conceptualization of the trial and the first draft of the manuscript. GR, KB, TL, and SF conducted the pretests. All authors were responsible for the drafting of this paper and approved the final manuscript.

## Pre-publication history

The pre-publication history for this paper can be accessed here:


